# 3-(2-Acetyl­anilino)propanoic acid

**DOI:** 10.1107/S1600536808035277

**Published:** 2008-11-13

**Authors:** Christopher R. Sparrow, Edwin H. Walker, Frank R. Fronczek

**Affiliations:** a Department of Chemistry, Southern University, Baton Rouge, LA 70813, USA; b Department of Chemistry, Louisiana State University, Baton Rouge, LA 70803-1804, USA

## Abstract

The title mol­ecule, C_11_H_13_NO_3_, has its propanoic acid group in an extended conformation, such that the mol­ecule is nearly planar, with a mean deviation of 0.036 Å [the maxima being 0.106 (1) and 0.110 (1) Å for the two methyl­ene C atoms]. The NH group forms an intra­molecular hydrogen bond with the acetyl group; in the crystal COOH group forms a centrosymmetric hydrogen-bonded dimer.

## Related literature

For general background, see: Crosby *et al.* (1961 ▶, 1962 ▶); Foley *et al.* (2003 ▶); Walker Jr *et al.* (2004 ▶); Yoshihara *et al.* (2001 ▶). For related structures, see: Slater *et al.* (2006 ▶). For hydrogen-bonding patterns, see: Etter (1990 ▶).
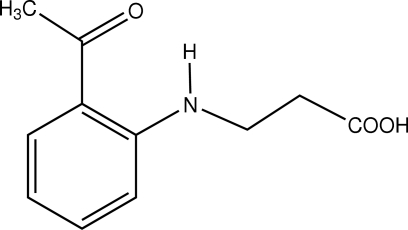


## Experimental

### 

#### Crystal data

C_11_H_13_NO_3_
*M* *_r_* = 207.22
Triclinic, 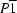

*a* = 5.1935 (10) Å
*b* = 9.8342 (16) Å
*c* = 9.920 (2) Å
α = 77.084 (12)°
β = 85.174 (11)°
γ = 83.019 (12)°
*V* = 489.32 (16) Å^3^
*Z* = 2
Mo *K*α radiation
μ = 0.10 mm^−1^
*T* = 90.0 (5) K
0.30 × 0.20 × 0.10 mm

#### Data collection

Nonius KappaCCD diffractometer with an Oxford Cryosystems Cryostream cooler
Absorption correction: none
11014 measured reflections
3012 independent reflections
2467 reflections with *I* > 2s(*I*)
*R*_int_ = 0.021

#### Refinement

*R*[*F* ^2^ > 2σ(*F* ^2^)] = 0.041
*wR*(*F* ^2^) = 0.113
*S* = 1.04
3012 reflections
143 parameters
H atoms treated by a mixture of independent and constrained refinement
Δρ_max_ = 0.39 e Å^−3^
Δρ_min_ = −0.29 e Å^−3^

### 

Data collection: *COLLECT* (Nonius, 2000 ▶); cell refinement: *SCALEPACK* (Otwinowski & Minor, 1997 ▶); data reduction: *DENZO* (Otwinowski & Minor, 1997 ▶) and *SCALEPACK*; program(s) used to solve structure: *SIR97* (Altomare *et al.*, 1999 ▶); program(s) used to refine structure: *SHELXL97* (Sheldrick, 2008 ▶); molecular graphics: *ORTEP-3 for Windows* (Farrugia, 1997 ▶); software used to prepare material for publication: *SHELXL97*.

## Supplementary Material

Crystal structure: contains datablocks global, I. DOI: 10.1107/S1600536808035277/pv2116sup1.cif

Structure factors: contains datablocks I. DOI: 10.1107/S1600536808035277/pv2116Isup2.hkl

Additional supplementary materials:  crystallographic information; 3D view; checkCIF report

## Figures and Tables

**Table 1:** Hydrogen-bond geometry (Å, °)

*D*—H⋯*A*	*D*—H	H⋯*A*	*D*⋯*A*	*D*—H⋯*A*
O2—H2*O*⋯O3^i^	0.909 (16)	1.747 (16)	2.6531 (11)	174.7 (14)
N1—H1*N*⋯O1	0.887 (14)	1.980 (14)	2.6690 (12)	133.4 (11)

Symmetry code: (i) 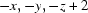.

**Fig. 1.**
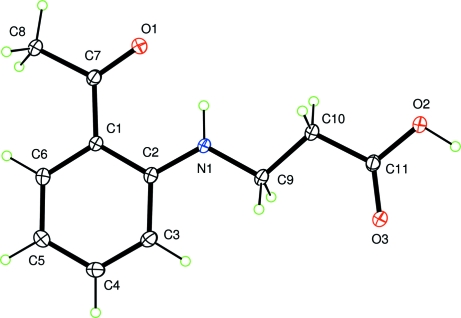
A view of the title compound; ellipsoids have been plotted at the 50% level and H atoms have been assigned arbitrary radii.

## supplementary crystallographic information

### Comment

Since the discovery that energy transfer from the triplet state of an organic ligand can efficiently sensitize the emissive states of metal ions (Crosby *et al.*, 1961; 1962) there has been considerable effort devoted to designing ligands that optimize this energy transfer and thus give efficient metal luminescence (Foley *et al.*, 2003). The radiationless energy transfer photoluminescence and/or electro-luminescence properties of aromatic carbonyl compounds are strongly affected by the presence of a substituent on the aromatic ring. In general, the π, π^*^ state is stabilized by introducing an electron-donating substituent on the aromatic ring, while the location of the n, π^*^ state is only slightly modified by the electron-donating substituent. Thus, the fluorescence properties of the compounds with close-lying ^1^(n, π^*^) and ^1^(π, π^*^) states are expected to depend markedly on the nature of the solvent, such as its polarity and hydrogen-bonding ability. In 2'-aminoacetophenone, the ^1^(n, π^*^) and ^1^(π, π^*^) states are closely located in the lowest excited singlet state because of the presence of a strong electron-donating substituent. Owing to the proximity of two electronic levels, the photophysical properties of 2'-aminoacetophenone is very sensitive to environment, such as solvent polarity and temperature (Yoshihara *et al.*, 2001). Therefore, the addition of a strong electron-donating substituent such as acrylic acid, which has the ability to form a solventless gel *via* hydrogen bonding, has been synthesized. The structure of the title compound, also known as 3-[(*N*-acetyl-phenyl)-azanediyl]-propionic acid, is herein described. Its synthesis takes advantage of the self-initiating condensation of 2'-aminoacetophenone with the vinyl group of the *ab* unsaturated acrylic acid *via* anti-Markovnikov addition, which is similar to chemistry involved in the synthesis of the novel 3,3',3"-nitrilotripropionic acid precursor gel that we have developed (Walker, *et al.*, 2004).

The structure of the molecule is shown in Figure 1. The acetyl group is nearly coplanar with the phenyl group, the C2—C1—C7—O2 torsion angle being -1.64 (15)°. The NH(CH_2_)_2_COOH substituent is extended, with torsion angles C1—C2—N1—C9 - 176.52 (9), C2—N1—C9—C10 - 172.10 (9), N1—C9—C10—C11 179.81 (8), and C9—C10—C11—O2 173.73 (8)°. Thus, the molecule does not differ greatly from planarity, with mean and maximum deviations given in the abstract. The geometry of the intramolecular hydrogen bond, graph set S(6), (Etter, 1990) is given in Table 1, and is quite similar to that found in *N*-(2-acetylphenyl)acetamide (Slater *et al.*, 2006). The COOH group forms a typical hydrogen bonded dimer of graph set *R*^2^_2_(8) about an inversion center, thus there are no extended networks of traditional hydrogen bonds.

### Experimental

A round bottom flask containing acrylic acid (1.051 g, 14.58 mmol) was stoppered and placed into the refrigerator overnight. In a similar round bottom flask, 2'-aminoacetophenoneHCl (1.0056 g, 0.79199 g of liberated 2-aminoacetophenone 5.861 mmol) was dissolved in 10 ml of deionized water and chilled overnight. The round bottom flask containing the cold acrylic acid was then placed into to an ice bath to maintain a temperature of approximately 273 K. The ice bath was used because the reaction of acrylic acid and 2'-aminoacetophenone was thought to be exothermic. The cold 2'-aminoacetophenone solution was slowly added with stirring to the cold acrylic acid. The mixture was allowed to sit in the ice bath for 30 minutes and then gradually warmed to room temperature, then kept at room temperature with continuous stirring overnight. A yellow precipitate was isolated by gravimetric filtration and washed with deionized water. The yellow solid material resulted in a yield of 0.8963 g (79.991%).

### Refinement

H atoms on C were placed in idealized positions with C—H distances 0.95 - 0.99 Å and thereafter treated as riding. Coordinates for the H atoms on N and O were refined. *U*_iso_ for H was assigned as 1.2 times *U*_eq_ of the attached atoms (1.5 for methyl and OH). A torsional parameter was refined for the methyl group.

### Crystal data

**Table:**

C_11_H_13_NO_3_	*Z* = 2
*M**_r_* = 207.22	*F*_000_ = 220
Triclinic, *P*1	*D*_x_ = 1.406 Mg m^−^^3^
Hall symbol: -P 1	Mo *K*α radiation λ = 0.71073 Å
*a* = 5.1935 (10) Å	Cell parameters from 2652 reflections
*b* = 9.8342 (16) Å	θ = 2.5–30.8º
*c* = 9.920 (2) Å	µ = 0.10 mm^−^^1^
α = 77.084 (12)º	*T* = 90.0 (5) K
β = 85.174 (11)º	Fragment, colorless
γ = 83.019 (12)º	0.30 × 0.20 × 0.10 mm
*V* = 489.32 (16) Å^3^	

### Data collection

**Table:**

Nonius KappaCCD diffractometer with an Oxford Cryosystems Cryostream cooler	2467 reflections with *I* > 2s(*I*)
Radiation source: fine-focus sealed tube	*R*_int_ = 0.021
Monochromator: graphite	θ_max_ = 30.8º
*T* = 90.0(5) K	θ_min_ = 2.6º
ω scans with κ offsets	*h* = −7→7
Absorption correction: none	*k* = −13→13
11014 measured reflections	*l* = −14→14
3012 independent reflections	

### Refinement

**Table:**

Refinement on *F*^2^	Secondary atom site location: difference Fourier map
Least-squares matrix: full	Hydrogen site location: inferred from neighbouring sites
*R*[*F*^2^ > 2σ(*F*^2^)] = 0.041	H atoms treated by a mixture of independent and constrained refinement
*wR*(*F*^2^) = 0.113	*w* = 1/[σ^2^(*F*_o_^2^) + (0.0573*P*)^2^ + 0.1339*P*] where *P* = (*F*_o_^2^ + 2*F*_c_^2^)/3
*S* = 1.04	(Δ/σ)_max_ < 0.001
3012 reflections	Δρ_max_ = 0.39 e Å^−^^3^
143 parameters	Δρ_min_ = −0.29 e Å^−^^3^
Primary atom site location: structure-invariant direct methods	Extinction correction: none

### Special details

**Table:**

Experimental. ^1^H (CDCl_3_): δ(p.p.m.) 9.02 (s, 1H), 7.78 (d, J = 8.0 Hz, 1H), 7.38 (t, J = 8.0 Hz, 1H), 6.74 (d, J = 8.0 Hz, 1H), 6.62 (t, J = 8.0 Hz, 1H), 3.58 (t, J = 7.0 Hz, 2H), 2.73 (t, J = 7.0 Hz, 2H), and 2.60 (s, 3H). ^13^C (CDCl_3_): δ (p.p.m.) 201.1, 176.7, 150.5, 135.2, 132.9, 117.9, 114.5, 111.3, 37.9, 33.8, and 27.9. IR (thin film, KBr plates, cm^-1^): 3297 (*m*), 2925 (w, br), 2881 (sh, w), 2635 (w), 2373 (w), 1698 (*s*), 1634 (*s*), 1569 (*m*), 1517 (*m*), 1430 (*m*), 1366 (sh), 1326(*m*), 1240 (*s*), 1156 (w), 1092(w), 1031(w), 943 (*m*), 825(w), 742 (*m*), 675(w), and 624 (w). Crystals were grown by evaporation from CDCl_3_.
Geometry. All e.s.d.'s (except the e.s.d. in the dihedral angle between two l.s. planes) are estimated using the full covariance matrix. The cell e.s.d.'s are taken into account individually in the estimation of e.s.d.'s in distances, angles and torsion angles; correlations between e.s.d.'s in cell parameters are only used when they are defined by crystal symmetry. An approximate (isotropic) treatment of cell e.s.d.'s is used for estimating e.s.d.'s involving l.s. planes.
Refinement. Refinement of *F*^2^ against ALL reflections. The weighted *R*-factor *wR* and goodness of fit *S* are based on *F*^2^, conventional *R*-factors *R* are based on *F*, with *F* set to zero for negative *F*^2^. The threshold expression of *F*^2^ > σ(*F*^2^) is used only for calculating *R*-factors(gt) *etc*. and is not relevant to the choice of reflections for refinement. *R*-factors based on *F*^2^ are statistically about twice as large as those based on *F*, and *R*- factors based on ALL data will be even larger.

### Fractional atomic coordinates and isotropic or equivalent isotropic displacement parameters (Å^2^)

**Table:**

	*x*	*y*	*z*	*U*_iso_*/*U*_eq_	
O1	0.96261 (15)	0.13504 (8)	0.35274 (7)	0.01656 (17)	
O2	0.30686 (15)	−0.07587 (8)	0.93327 (7)	0.01593 (17)	
H2O	0.183 (3)	−0.0849 (15)	1.0039 (16)	0.024*	
O3	0.04143 (14)	0.11745 (8)	0.85415 (7)	0.01599 (17)	
N1	0.53905 (17)	0.21868 (9)	0.49711 (8)	0.01437 (18)	
H1N	0.673 (3)	0.1541 (14)	0.4924 (13)	0.017*	
C1	0.70722 (18)	0.35189 (10)	0.27655 (10)	0.01194 (19)	
C2	0.51897 (19)	0.33391 (10)	0.39115 (10)	0.01242 (19)	
C3	0.3083 (2)	0.43948 (11)	0.39170 (10)	0.0161 (2)	
H3	0.1787	0.4282	0.4657	0.019*	
C4	0.2866 (2)	0.55873 (11)	0.28701 (11)	0.0168 (2)	
H4	0.1438	0.6283	0.2906	0.020*	
C5	0.4725 (2)	0.57819 (11)	0.17590 (11)	0.0165 (2)	
H5	0.4581	0.6605	0.1043	0.020*	
C6	0.6778 (2)	0.47510 (10)	0.17238 (10)	0.0145 (2)	
H6	0.8038	0.4879	0.0967	0.017*	
C7	0.92650 (19)	0.24478 (10)	0.26361 (10)	0.01281 (19)	
C8	1.1133 (2)	0.26868 (11)	0.13707 (10)	0.0157 (2)	
H8A	1.2116	0.3472	0.1386	0.024*	
H8B	1.0155	0.2905	0.0532	0.024*	
H8C	1.2337	0.1838	0.1374	0.024*	
C9	0.34277 (19)	0.19459 (11)	0.61054 (10)	0.0142 (2)	
H9A	0.1798	0.1767	0.5757	0.017*	
H9B	0.3060	0.2789	0.6508	0.017*	
C10	0.43852 (19)	0.06928 (10)	0.72115 (10)	0.01378 (19)	
H10A	0.4756	−0.0145	0.6801	0.017*	
H10B	0.6025	0.0875	0.7548	0.017*	
C11	0.24229 (19)	0.04048 (10)	0.84099 (10)	0.01244 (19)	

### Atomic displacement parameters (Å^2^)

**Table:**

	*U*^11^	*U*^22^	*U*^33^	*U*^12^	*U*^13^	*U*^23^
O1	0.0166 (4)	0.0146 (3)	0.0149 (3)	0.0019 (3)	0.0026 (3)	0.0012 (3)
O2	0.0148 (4)	0.0162 (4)	0.0127 (3)	0.0014 (3)	0.0038 (3)	0.0020 (3)
O3	0.0152 (4)	0.0162 (4)	0.0132 (3)	0.0019 (3)	0.0041 (3)	0.0003 (3)
N1	0.0136 (4)	0.0144 (4)	0.0111 (4)	0.0025 (3)	0.0048 (3)	0.0014 (3)
C1	0.0110 (4)	0.0123 (4)	0.0114 (4)	−0.0009 (3)	0.0016 (3)	−0.0012 (3)
C2	0.0127 (4)	0.0134 (4)	0.0104 (4)	−0.0015 (3)	0.0016 (3)	−0.0018 (3)
C3	0.0145 (5)	0.0179 (5)	0.0141 (4)	0.0014 (4)	0.0030 (3)	−0.0027 (4)
C4	0.0160 (5)	0.0150 (5)	0.0177 (5)	0.0030 (4)	0.0004 (4)	−0.0030 (4)
C5	0.0174 (5)	0.0135 (4)	0.0162 (5)	0.0002 (4)	0.0000 (4)	0.0005 (3)
C6	0.0152 (4)	0.0145 (4)	0.0125 (4)	−0.0021 (4)	0.0021 (3)	−0.0006 (3)
C7	0.0115 (4)	0.0146 (4)	0.0118 (4)	−0.0016 (3)	0.0015 (3)	−0.0024 (3)
C8	0.0146 (4)	0.0162 (5)	0.0136 (4)	0.0001 (4)	0.0045 (3)	−0.0005 (3)
C9	0.0126 (4)	0.0169 (5)	0.0109 (4)	−0.0001 (3)	0.0033 (3)	−0.0008 (3)
C10	0.0126 (4)	0.0155 (4)	0.0113 (4)	−0.0007 (3)	0.0028 (3)	−0.0005 (3)
C11	0.0132 (4)	0.0130 (4)	0.0107 (4)	−0.0022 (3)	0.0015 (3)	−0.0020 (3)

### Geometric parameters (Å, °)

**Table:**

O1—C7	1.2384 (12)	C4—H4	0.9500
O2—C11	1.3228 (12)	C5—C6	1.3829 (14)
O2—H2O	0.909 (16)	C5—H5	0.9500
O3—C11	1.2278 (12)	C6—H6	0.9500
N1—C2	1.3628 (12)	C7—C8	1.5135 (13)
N1—C9	1.4519 (12)	C8—H8A	0.9800
N1—H1N	0.887 (14)	C8—H8B	0.9800
C1—C6	1.4075 (13)	C8—H8C	0.9800
C1—C2	1.4299 (13)	C9—C10	1.5193 (14)
C1—C7	1.4722 (14)	C9—H9A	0.9900
C2—C3	1.4145 (14)	C9—H9B	0.9900
C3—C4	1.3819 (14)	C10—C11	1.4993 (13)
C3—H3	0.9500	C10—H10A	0.9900
C4—C5	1.3983 (15)	C10—H10B	0.9900
			
C11—O2—H2O	108.7 (9)	O1—C7—C8	118.76 (9)
C2—N1—C9	122.44 (8)	C1—C7—C8	119.13 (8)
C2—N1—H1N	117.8 (9)	C7—C8—H8A	109.5
C9—N1—H1N	119.7 (9)	C7—C8—H8B	109.5
C6—C1—C2	118.53 (9)	H8A—C8—H8B	109.5
C6—C1—C7	119.47 (9)	C7—C8—H8C	109.5
C2—C1—C7	122.00 (8)	H8A—C8—H8C	109.5
N1—C2—C3	120.39 (9)	H8B—C8—H8C	109.5
N1—C2—C1	121.56 (9)	N1—C9—C10	109.68 (8)
C3—C2—C1	118.05 (9)	N1—C9—H9A	109.7
C4—C3—C2	121.48 (9)	C10—C9—H9A	109.7
C4—C3—H3	119.3	N1—C9—H9B	109.7
C2—C3—H3	119.3	C10—C9—H9B	109.7
C3—C4—C5	120.76 (9)	H9A—C9—H9B	108.2
C3—C4—H4	119.6	C11—C10—C9	111.66 (8)
C5—C4—H4	119.6	C11—C10—H10A	109.3
C6—C5—C4	118.62 (9)	C9—C10—H10A	109.3
C6—C5—H5	120.7	C11—C10—H10B	109.3
C4—C5—H5	120.7	C9—C10—H10B	109.3
C5—C6—C1	122.54 (9)	H10A—C10—H10B	107.9
C5—C6—H6	118.7	O3—C11—O2	122.63 (9)
C1—C6—H6	118.7	O3—C11—C10	123.78 (9)
O1—C7—C1	122.11 (9)	O2—C11—C10	113.59 (8)
			
C9—N1—C2—C3	3.20 (15)	C2—C1—C6—C5	−0.84 (15)
C9—N1—C2—C1	−176.52 (9)	C7—C1—C6—C5	178.13 (10)
C6—C1—C2—N1	−178.48 (9)	C6—C1—C7—O1	179.42 (9)
C7—C1—C2—N1	2.57 (15)	C2—C1—C7—O1	−1.64 (15)
C6—C1—C2—C3	1.80 (14)	C6—C1—C7—C8	−0.98 (14)
C7—C1—C2—C3	−177.15 (9)	C2—C1—C7—C8	177.96 (9)
N1—C2—C3—C4	178.57 (10)	C2—N1—C9—C10	−172.10 (9)
C1—C2—C3—C4	−1.71 (15)	N1—C9—C10—C11	179.81 (8)
C2—C3—C4—C5	0.58 (16)	C9—C10—C11—O3	−6.99 (14)
C3—C4—C5—C6	0.45 (16)	C9—C10—C11—O2	173.73 (8)
C4—C5—C6—C1	−0.30 (16)		

### Hydrogen-bond geometry (Å, °)

**Table:**

*D*—H···*A*	*D*—H	H···*A*	*D*···*A*	*D*—H···*A*
O2—H2O···O3^i^	0.909 (16)	1.747 (16)	2.6531 (11)	174.7 (14)
N1—H1N···O1	0.887 (14)	1.980 (14)	2.6690 (12)	133.4 (11)

Symmetry codes: (i) −*x*, −*y*, −*z*+2.
